# Prediction of the mechanical properties of zeolite pellets for aerospace molecular decontamination applications

**DOI:** 10.3762/bjnano.7.169

**Published:** 2016-11-18

**Authors:** Guillaume Rioland, Patrick Dutournié, Delphine Faye, T Jean Daou, Joël Patarin

**Affiliations:** 1 Université de Strasbourg (UDS), Université de Haute-Alsace (UHA), CNRS, Equipe Matériaux à Porosité Contrôlée (MPC), Institut de Science des Matériaux de Mulhouse (IS2M), UMR 7361, 3 bis rue Alfred Werner, F-68093 Mulhouse, France; 2 Service Laboratoires & Expertise, Centre National d’Etudes Spatiales (CNES), 18 avenue Edouard Belin, 31401 Toulouse Cedex 9, France

**Keywords:** design of experiments methodology, mechanical performance, molecular decontamination, operating optimums, zeolite pellets

## Abstract

Zeolite pellets containing 5 wt % of binder (methylcellulose or sodium metasilicate) were formed with a hydraulic press. This paper describes a mathematical model to predict the mechanical properties (uniaxial and diametric compression) of these pellets for arbitrary dimensions (height and diameter) using a design of experiments (DOE) methodology. A second-degree polynomial equation including interactions was used to approximate the experimental results. This leads to an empirical model for the estimation of the mechanical properties of zeolite pellets with 5 wt % of binder. The model was verified by additional experimental tests including pellets of different dimensions created with different applied pressures. The optimum dimensions were found to be a diameter of 10–23 mm, a height of 1–3.5 mm and an applied pressure higher than 200 MPa. These pellets are promising for technological uses in molecular decontamination for aerospace-based applications.

## Introduction

Molecular contamination is of great concern in aerospace-based applications. Once satellites are in orbit, molecules contained in paints, adhesives or glues can outgas and form films or droplets on the surface of mirrors, lenses, solar cells or thermal detectors. The National Aeronautic and Space Administration (NASA) has investigated the chemical nature of these molecules; hydrocarbons and plasticizers were identified as the most important of the outgassed molecules [1–2]. The surface of all the on-board equipment such as the thermal captor or optics could become covered by molecules, leading to a deterioration of these tools.

Porous alumina, silica, zeolites and charcoal have been tested to solve the molecular contamination issue, and zeolites have been selected as the best candidates. At very low concentration, zeolites are able to trap water and volatile organic compounds thanks to their great adsorption abilities [3]. The French Space Agency (CNES) studied zeolites for the adsorption of volatile organic compounds in satellites [4–8].

Zeolites are aluminosilicate materials with micropores. They are the result of a combination of AlO_4_ and SiO_4_ tetrahedra. Zeolites are often used in adsorption and catalytic applications due to their high thermal stability and interesting adsorption properties [9–15]. Today, 231 different zeolite structures have been discovered, but only some of them are currently used in industry [16–19].

In most applications, and for more convenience (i.e., handling, transport), zeolites cannot be used in powder-form. For space-based applications, particulate contamination has to be avoided. However, binders are necessary to improve the mechanical properties and to allow for the compression of powders. In the literature, significant amounts of binder is used to shape zeolites, leading to objects (e.g., cylinders, beads) with high mechanical resistance and optimal dimensions [20–24]. In the literature, the use of a hydraulic press to form zeolite pellets is not common, although some articles have been published [24–25]. Moreover, the mechanical resistance (compression tests) is either low (between 1 and 10 MPa) or the amount of binder is very high (more than 20 wt %) [20–25]. In our previous work, a hydraulic press was used to make zeolite pellets with very good mechanical resistance (about 100 MPa) and with a very small amount of methylcellulose or sodium metasilicate as binder (5 wt %) [8]. Because vibrations or impact upon satellites launch could cause the destruction of these bodies and contaminate on-board equipment, improved mechanical performance of zeolite materials is highly sought after. In order to use zeolite pellets for space-based applications, they must have both good mechanical properties and adhere to size limitations (e.g., volume, diameter, height and number of pellets).

Recently, Lai et al. developed a new empirical model able to predict the mechanical properties (ultimate compressive strength) of a binary mixture pellet based on the volume fraction of HZSM-5 (MFI) zeolite in the binary pellet, the hold time and elastic rebound [26].

In this paper, we propose two other models able to predict the ultimate compressive strength of zeolite pellets regardless of dimension. These models (used in many fields) are based on a design of experiments (DOE) methodology [27–29]. Thus, the optimum dimensions will be linked to the best mechanical resistance, leading to an optimum pellet able to trap pollutants without being damaged by impact or vibrations.

## Experimental

### Materials

Two hydrophilic and two hydrophobic zeolites have been chosen to make zeolite pellets:

- FAU 13X powder with a crystal size of 3–5 µm and a Si/Al molar ratio of 1.2 was purchased from Sigma-Aldrich.

- Na-LTA powder with a crystal size of 3–5 µm and a Si/Al molar ratio of 1.1 was purchased from CECA.

- MFI Sicade-2 powder with a crystal size of 3–5 µm and an infinite Si/Al molar ratio was purchased from Zephir Alsace.

- BEA powder with a crystal size of 3–5 µm and a Si/Al molar ratio of 136 was synthesized according to the protocol described below.

The gel preparation and hydrothermal synthesis were performed using a polypropylene bottle. First, 1.5 g of hydrofluoric acid (Sigma-Aldrich, 40% in water) was poured on 11 g of tetraethylammonium hydroxide (Sigma-Aldrich, 40% in water) used as structure-directing agent. 0.12 g of BEA zeolite germs are added and blended under vigorous mixing (700 rpm). 6 g of silica (Zeosil^®^ 1165 MP, Rhodia) were finally added, giving the following gel molar composition: 1:0.3:0.3:4.17 SiO_2_/(C_2_H_5_)_4_NOH/HF/H_2_O. The homogenization is carried out with a Teflon^®^ rod.

The gel was introduced into PTFE-lined stainless-steel autoclaves and heated at 150 °C for 2 days. After synthesis the product was filtered, washed with deionized water several times and dried overnight at 100 °C. The organic compounds were finally removed by calcination at 550 °C for 8 h in air (ramp at 1 °C·min^−1^).

Methylcellulose (MC) was purchased from Acros Organics. Anhydrous sodium metasilicate (Na_2_SiO_3_) was purchased from Fluka Chemicals.

### Characterization techniques

The ultimate compressive strength of the consolidated pellets was determined by uniaxial and diametric compression tests. The stress and displacement were recorded until the pellets crack by applying a displacement rate of 0.5 mm/min with an Instron 4505 Zwick dynamometer. To obtain a statistically significant data distribution, three identical pellets were analyzed for each parameter.

### Preparation of zeolite pellets

In a recent paper, the preparation of MFI-type zeolite pellets was described [8]. The pelletization apparatus is divided into two parts: a manual hydraulic press (Atlas 15T Manual Hydraulic Press, Specac) and a pellet die where the powder is incorporated. The zeolite pellets were prepared from a mixture of binder, 1:4 zeolites (FAU, BEA, LTA and MFI-types) to water. First, distilled water was mixed with a certain amount of binder (5 wt % of the pellet for optimized conditions). This amount of water was optimized: if too much water is added, the mixtures cannot be pelletized (the mixture is liquid). On the other hand, with too little binder, cracks appear after the ejection of pellets. Then, zeolite is added. Water improves the diffusion of binder in zeolite powder and allows the formation of a MC or Na_2_SiO_3_ gel which acts as glue that holds particles together. The mixture was stirred and put in the pellet die for pelletization. A definite compression load was applied during 5 min in order to form a pellet. The pellets were finally dried at 70 °C during 24 h.

### Calculation and experimental section

To predict the mechanical resistance of zeolite pellets of various dimensions, it is necessary to know the ultimate compressive strength applied to a mixture composed of one to four zeolites (FAU, BEA, LTA and MFI-types) and 5 wt % of binder (methylcellulose or sodium metasilicate).

This ultimate compressive strength depends on some factors, such as the compression load and the dimensions of the pellet. These dimensions are linked to the amount of the mixture introduced in the pellet die. The mechanical properties of two different formulations compacted with the same compression loads and the same diameter will be different because the pellet height is not the same. The pressure applied to the mixture is assumed as the applied force (weight) divided by the surface of the pellet. The ultimate compressive strength was determined by uniaxial and diametric compression tests.

To analyse the influence of the three studied parameters on the mechanical properties, a parametric study of the ultimate compressive strength was conducted by using DOE. This methodology allows the maximum information to be obtained about the operating parameter influencing the process while minimizing the number of experimental tests [30]. In this case, the influence of three parameters (diameter, height and applied pressure) on the ultimate compressive strength of the final pellet is studied.

Table 1 shows the experimental tests that have been used for conception of our mathematical models. The studied range is *e* = 1 to 7 mm (height), Ø = 10 to 32 mm (diameter) and *P* = 29.5 to 277.5 MPa (applied pressure).

**Table 1:** Experimental tests (3 or 6 repeated tests) to study the influence of three parameters (diameter, height and applied pressure) on the ultimate compressive strengths of the final pellets. *Y* is the uniaxial ultimate compressive strength and *Z* is the diametric ultimate compressive strength.

Dimensions (diameter × height) (mm × mm)	Applied pressure, *P* (MPa)	*Y* (MPa)	*Z* (MPa)

13.0 × 1.0	153.5	70.0, 50.0, 60.0	0.80, 0.82, 0.81
13.0 × 2.0	110.0 230.0	44.0, 48.0, 41.0 75.0, 71.0, 78.0	0.70, 0.75, 0.71 0.90, 0.85, 0.86
13.0 × 4.0	29.5 150.0 153.5^a^ 270.0 277.5^a^	5.0, 4.5, 6.5 21.0, 21.0, 17.0 20.0, 18.0, 22.0, 23.5, 20.5, 22.5 45.0, 50.0, 53.0 45.0, 50.0, 42.0, 51.0, 50.0, 45.0	0.10, 0.08, 0.07 0.39, 0.45, 0.44 0.59, 0.65, 0.66, 0.61, 0.69, 0.70 1.10, 1.05, 1.09 1.10, 1.09, 1.05, 1.10, 1.08, 1.07
13.0 × 6.0	110.0 230.0	14.0, 11.0, 12.0 21.0, 23.0, 19.0	0.48, 0.55, 0.44 0.99, 0.91, 0.90
13.0 × 7.0	153.5^a^	11.0, 9.0, 10.0, 8.0, 7.0, 8.0	0.60, 0.64, 0.68, 0.60, 0.69, 0.69
10.0 × 2.5	29.5 ^a^ 91.5 ^a^ 215.5	7.5, 7, 5.5, 5, 7.0, 8.0 15.0, 14.0, 18.0, 15.0, 17.0, 18.0 45.0, 41.0, 37.0	0.25, 0.34, 0.21, 0.28, 0.30, 0.31 0.70, 0.67, 0.68, 0.71, 0.69, 0.64 1.30, 1.35, 1.25
10.0 × 5.5	29.5 91.5^a^ 215.5	2.5, 2.8, 2.0 4.5, 5.0, 5.0, 3.5, 3.0, 5.5 8.0, 9.0, 11.0	0.15, 0.18, 0.11 0.30, 0.37, 0.38, 0.38, 0.36, 0.40 1.10, 1.15, 1.05
32.0 × 2.0	110.0 230.0	60.0, 58.0, 63.0 120.0, 111.0, 123.0	0.18, 0.15, 0.20 0.45, 0.51, 0.55
32.0 × 2.5	91.5 215.5^a^	45.0, 50.0, 55.0 95.0, 90.0, 85.0, 100.0, 90.0, 95.0	0.12, 0.11, 0.13 0.40, 0.37, 0.35, 0.41, 0.38, 0.41
32.0 × 4.0	150.0	56.0, 60.0, 61.0	0.18, 0.22, 0.20
32.0 × 5.5	91.5^a^ 215.5	40.0, 38.0, 30.0, 40.0, 35.0, 48.0 80.0, 85.0, 80.0	0.10, 0.10, 0.08, 0.11, 0.07, 0.09 0.29, 0.24, 0.31
32.0 × 6.0	110.0 230.0	45.0, 40.0, 41.0 84.0, 90.0, 80.0	0.15, 0.19, 0.16 0.34, 0.36, 0.35
32.0 × 7.0	230.0	91.0, 95.0, 88.0	0.24, 0.26, 0.21

^a^For some conditions, 6 mechanicals tests have been carried out.

The studied parameters of each test are chosen to optimize the pertinence and the relevance of the experimental tests. These parameters are normalized to compare the effects and the influence of the studied function. A rotatable central composite design was used and consists of three distinct sets of experimental runs: a set of central points, a full factorial design (orthogonal) and a set of axial points identical to the central point except for one parameter (values outside the studied range) [28,31]. All the experimental tests are systematically repeated 3 times.

To explore relationships between the studied function and the operating parameters a surface response methodology was used. A second-degree polynomial equation including interactions was used to approximate experimental results. A set of 105 equations (corresponding to the experimental trials) with 10 coefficients was statically solved by minimizing a quadratic criterion.

## Results and Discussion

### Uniaxial compression tests

**Figure 1:**
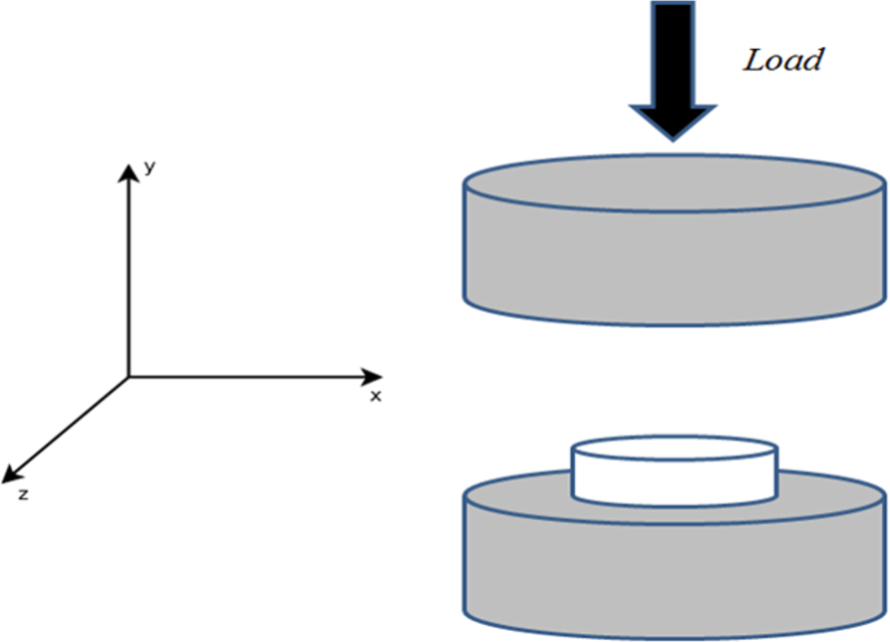
Schematic of uniaxial compression test of a zeolite pellet along the *y*-axis.

This test is carried out along the *y*-axis as shown in Figure 1. Experimental, ultimate compressive strengths were investigated by using the DOE methodology [32]. As mentioned before, the studied parameters have been described.

The first model is estimated from results of the uniaxial compression tests by minimizing the quadratic criterion between experimental and numerical results. The obtained model is:

[1]
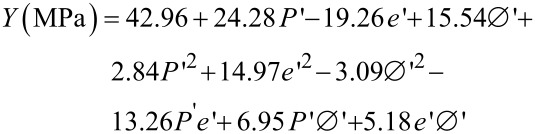


where *Y* is the ultimate compressive strength (MPa) and *P*’, *e*’, Ø’ are the values of the applied pressure (MPa), the height (mm) and the diameter (mm) of the pellet, respectively, normalized in the range (−1,1).

The maximal error calculated with the 105 experimental tests is less than 12 MPa and it is less than the maximal experimental error (13 MPa) observed for the repeated tests.

This equation shows that the ultimate compressive strength linearly depends on the applied pressure and the diameter. Indeed, quadratic terms of the diameter and the applied pressure are small compared to the linear parameters. The pertinence of the mathematical approximation is investigated for three series of six experimental tests. The results are summarized in Table 2.

**Table 2:** Experimental and calculated ultimate compressive strengths for 3 series of 6 experimental tests (uniaxial compression tests). *Y*_exp_ is the experimental and *Y* the calculated ultimate compressive strengths of the pellets, respectively.

Series 1 (Ø = 10 mm, *P* = 91.5 MPa, *e* = 2.4 ± 0.1 mm)						

*Y*_exp_ (MPa)	15	14	18	15	17	18
*Y* (MPa)	16.7					

Series 2 (Ø = 13 mm, *P* = 277.5 MPa, *e* = 4.1 ± 0.2 mm)						

*Y*_exp_ (MPa)	45	50	42	51	50	45
*Y* (MPa)	47.2					

Series 3 (Ø = 32 mm, *P* = 91.5 MPa, *e* = 5.5 ± 0.4 mm)						

*Y*_exp_ (MPa)	40	38	30	40	35	48
*Y* (MPa)	39.0					

Each series corresponds to a particular pellet diameter (10, 13 and 32 mm, respectively). The observed maximal error is all the more significant as the pellet diameter is increased. The average experimental ultimate compressive strengths are 16.2 ± 2.2 MPa, 47.2 ± 5.2 MPa and 38.5 ± 9.5 MPa for pellet diameters of 10, 13 and 32 mm, respectively. The average experimental values are very close to the calculated values obtained by using Equation 1 (16.7, 47.2 and 39.0, respectively).

**Figure 2:**
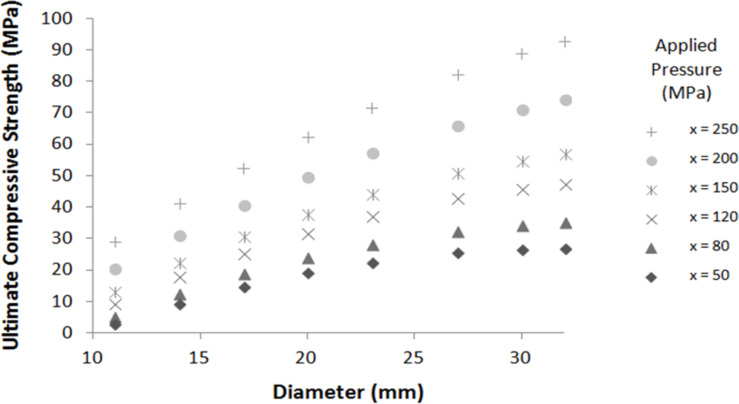
Ultimate compressive strength calculated by using Equation 1 for different pellet diameters and applied pressures (pellet height = 4.5 mm).

Table 2 confirms that the model can be used for these zeolite pellets: the calculated ultimate compressive strengths fit with the experimental ultimate compressive strengths, even when the applied pressures are out of the domain previously selected. The approximation model is now confirmed with the different experimental pellets and can be used for any operating conditions included in the studied range for predictive calculations. Figure 2 shows the ultimate compressive strength function of the pellet diameter and the applied pressure for a pellet height equal to 4.5 mm. These results show that the ultimate compressive strength increases when diameter and the applied pressure increase.

**Figure 3:**
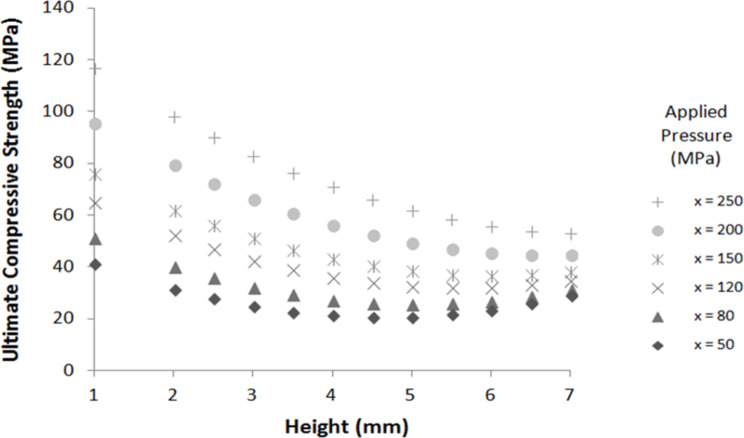
Ultimate compressive strength calculated by using Equation 1 for different pellet heights and applied pressures (pellet diameter = 21 mm).

The results obtained with Equation 1 for different pellet heights are shown in Figure 3 for a pellet diameter of 21 mm. The ultimate compressive strength is maximal for thin pellets and pelletized at high pressure. Small-height and large-diameter pellets compacted at high pressures show the best mechanical properties in terms of uniaxial compression resistance.

**Figure 4:**
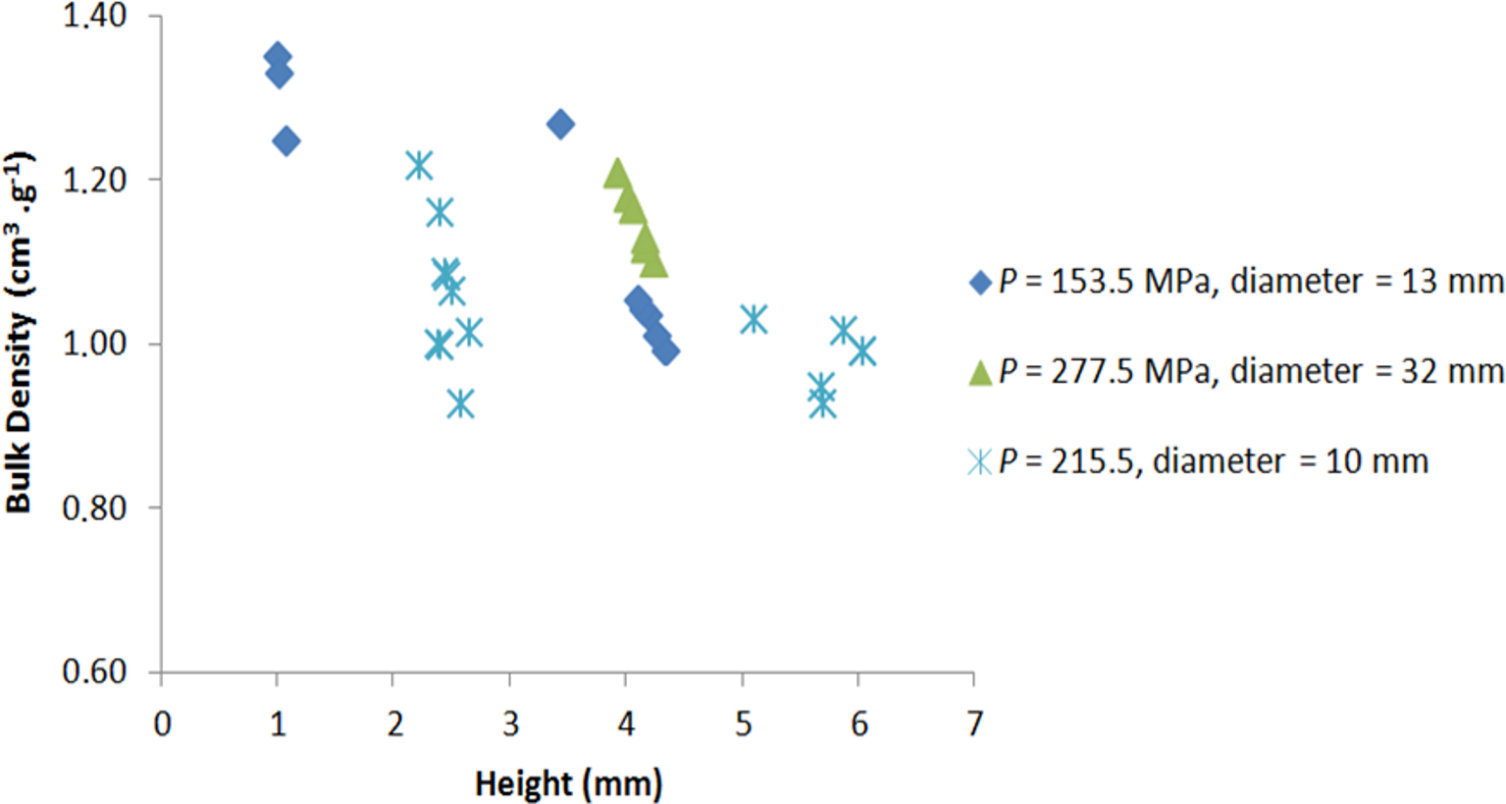
Bulk pellet density as a function of pellet height for different applied pressures. The diameters of the studied pellets are 10, 13 and 32 mm for applied pressures of 215.5, 153.5 and 277.5 MPa, respectively.

These results can be partially explained by the inhomogeneity of the density in the thickness of the material, especially for thicker pellets. Figure 4 shows the pellet density for different pellet heights and diameter. The diameter of the studied pellets are 10, 13 and 32 mm for applied pressures of 215.5, 153.5 and 277.5 MPa, respectively.

The density is higher for thin pellets and decreases with the pellet size. This rapid density depletion for thin pellets (height between 1 and 3 mm) seems to level off for thick pellets. This observation can be explained by the formation of two dense layers on both sides of the pelletized material, creating a density gradient in the material. This can also explain the results presented in Figure 3. Indeed, the ultimate compressive strength is higher for thin pellets because they are denser materials [33].

**Figure 5:**
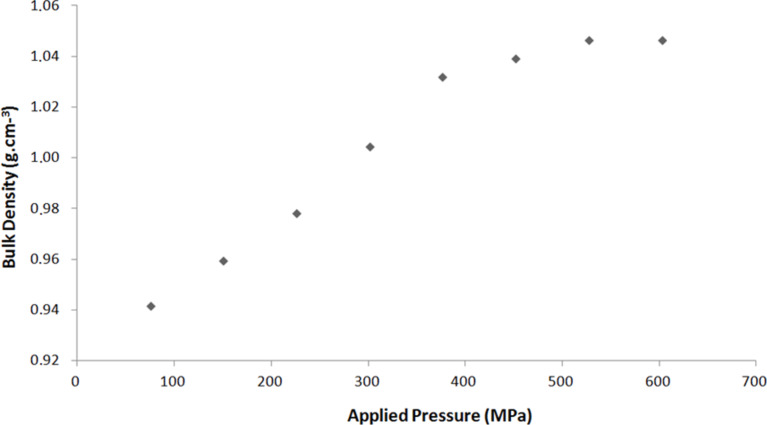
Bulk density as a function of the pressure applied to the mixture for 13 × 3 mm^2^ pellets made with 5 wt % of binder. The pellets may be comprised of one to four zeolites.

To confirm this observation, different measures of densities are shown in Figure 5 as a function of the applied pressure. The density of pellets pelletized at 277.5 MPa are higher than those pelletized at 153.5 MPa (for the same diameter and height). The correlation between samples size (diameter and height) and mechanical properties makes physical sense. Indeed, when the applied pressure (compression load) increases, the bulk density of the pellet increases. For example, the pellet height decreases from 3.2 to 2.8 mm when the applied pressure increases from 75 to 600 MPa with the same mass of zeolite (0.4 g, diameter = 13 mm). This behavior is linear up to 400 MPa (studied range) and after 400 MPa the density of the pellet levels off (1050 kg·m^−3^). This can be explained by a density gradient along the pellet height. The thicker the pellet, the higher the density gradient is in the material [33].

### Diametric compression tests

**Figure 6:**
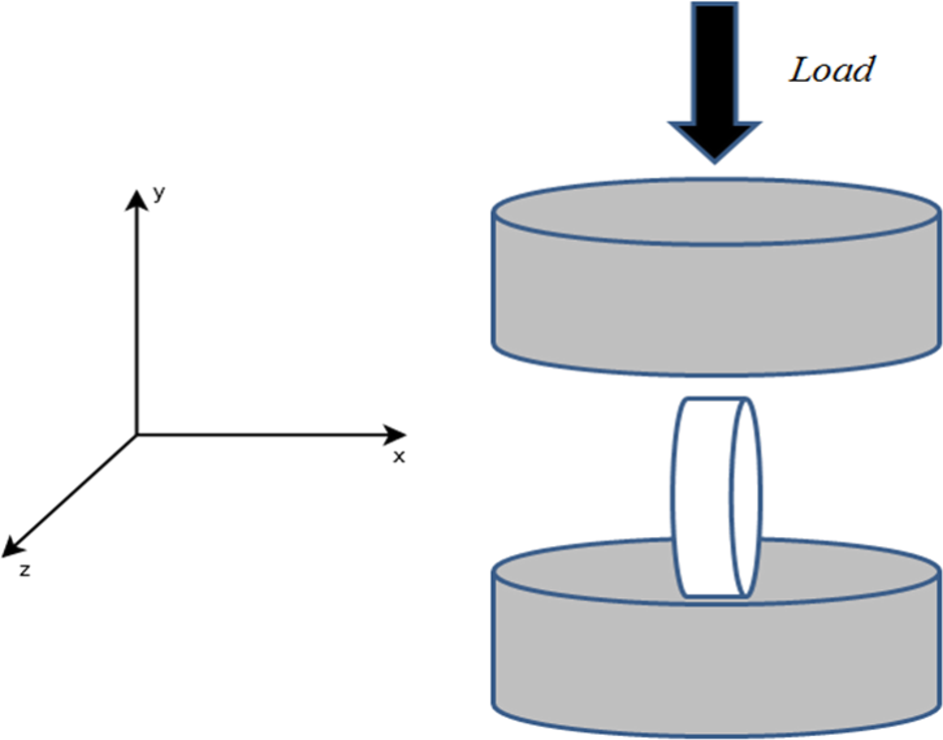
Schematic of diametric compression test of a zeolite pellet along the *y*-axis.

A second mechanical test was performed to enhance the knowledge about the pellet mechanical stability [32]. This test is carried out along the *y-*axis as shown in Figure 6, where the pellet is now shown on its edge. This test is commonly called the “Brazilian test”. Experimental ultimate compressive strengths are studied according to pellet size and the applied pressure.

As this was performed for uniaxial compression tests, experimental results of diametric compression tests are numerically approximated by a second order equation according to the DOE methodology. The best-fit parameters are obtained by minimizing a quadratic criterion. The obtained model is:

[2]
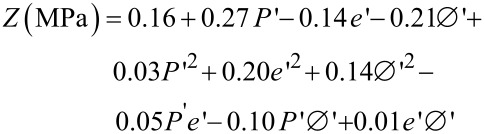


where *Z* is the ultimate compressive strength (MPa) and *P*’, *e*’, Ø’ are the values of the applied pressure (MPa), the height (mm) and the diameter (mm) of the pellet, respectively, normalized in the range (−1,1).

The investigation is performed with 105 experimental tests and the maximal error was less than 0.14 MPa. The pertinence of the mathematical approximation is investigated for three series of 6 experimental tests. The results are summarized in Table 3.

**Table 3:** Experimental and calculated ultimate compressive strengths for 3 series of 6 experimental tests (diametric compression tests). *Z*_exp_ is the experimental and *Z* the calculated ultimate compressive strengths of the pellets, respectively.

Series 1 (Ø = 10 mm, *P* = 91.5 MPa, *e* = 2.4 ± 0.2 mm)						

*Z*_exp_ (MPa)	0.70	0.67	0.68	0.71	0.74	0.69
*Z* (MPa)	0.68					

Series 2 (Ø = 13 mm, *P* = 277.5 MPa, *e* = 4.1 ± 0.1 mm)						

*Z*_exp_ (MPa)	1.10	1.05	1.09	1.10	1.05	1.09
*Z* (MPa)	1.06					

Series 3 (Ø = 32 mm, *P* = 91.5 MPa, *e* = 5.6 ± 0.2 mm)						

*Z*_exp_ (MPa)	0.09	0.10	0.08	0.11	0.07	0.09
*Z* (MPa)	0.05					

Each series corresponds to a particular pellet diameter (10, 13 and 32 mm, respectively). The observed maximal error is all the more significant as the pellet is larger in diameter (Series 3). The average experimental ultimate compressive strengths are 0.69 ± 0.05 MPa, 1.08 ± 0.03 MPa and 0.09 ± 0.02 MPa for pellet diameters of 10, 13 and 32 mm, respectively. Nevertheless, given the low strength values, the experimental relative error is significant (around 22% for Series 3). The calculated values obtained by using Equation 2 give a good approximation of experimental results in the studied range.

**Figure 7:**
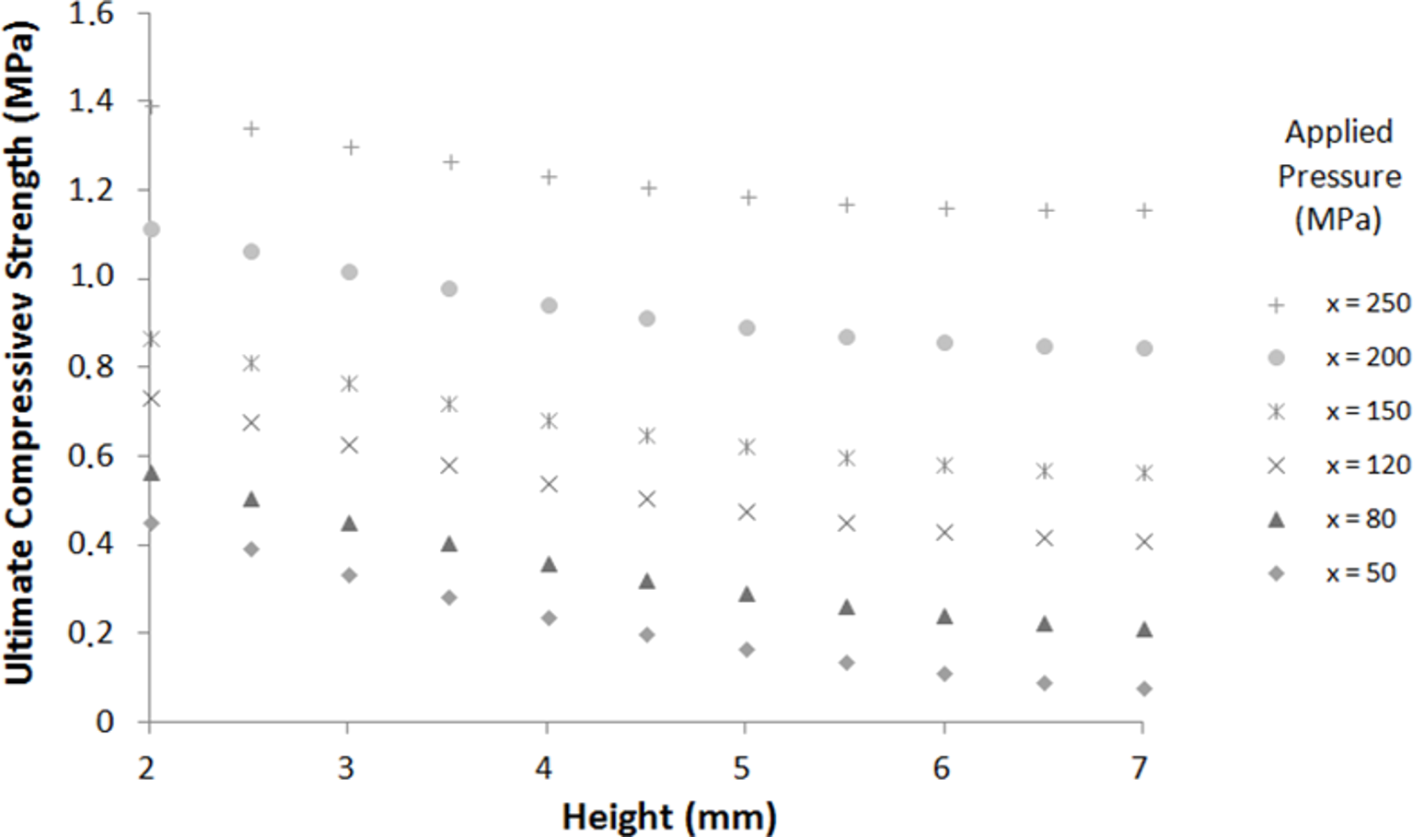
Ultimate compressive strength calculated by using Equation 2 as a function of pellet height for different applied pressures (pellet diameter = 10 mm).

The analysis of the estimated parameters (coefficients of Equation 2) shows that the highest mechanical strengths are obtained for thin and compact pellets pelletized at high pressure. These results exhibit the same trend as the previous ones; indeed, the best mechanical properties are obtained with dense pellets. Figure 7 shows the calculated ultimate strength function of pellet height and applied pressure for a pellet of 10 mm diameter. The ultimate compressive strength increases when the applied pressure increases and when the pellet height decreases.

These outcomes confirm that the model can be used for a predictive purpose to simulate the mechanical strength of zeolite pellets. The calculated ultimate compressive strengths fit with the experimental ultimate compressive strengths in the studied range for diametric compression operations.

### Simultaneous analysis of mechanical tests and operating optimums

In order to use zeolite pellets for space-based applications, both mechanical properties and size limitations (volume, diameter, height and number of pellets) must be met. Therefore, each mechanical test was separately described by a three-dimensional equation of degree two as follows:

[3]
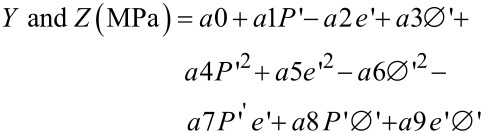


where *Y* and *Z* are the ultimate compressive strengths (MPa) and *P*’, *e*’, Ø’ are the values of the applied pressure (MPa), the height (mm) and the diameter (mm) of the pellet, respectively, normalized in the range (−1,1).

**Figure 8:**
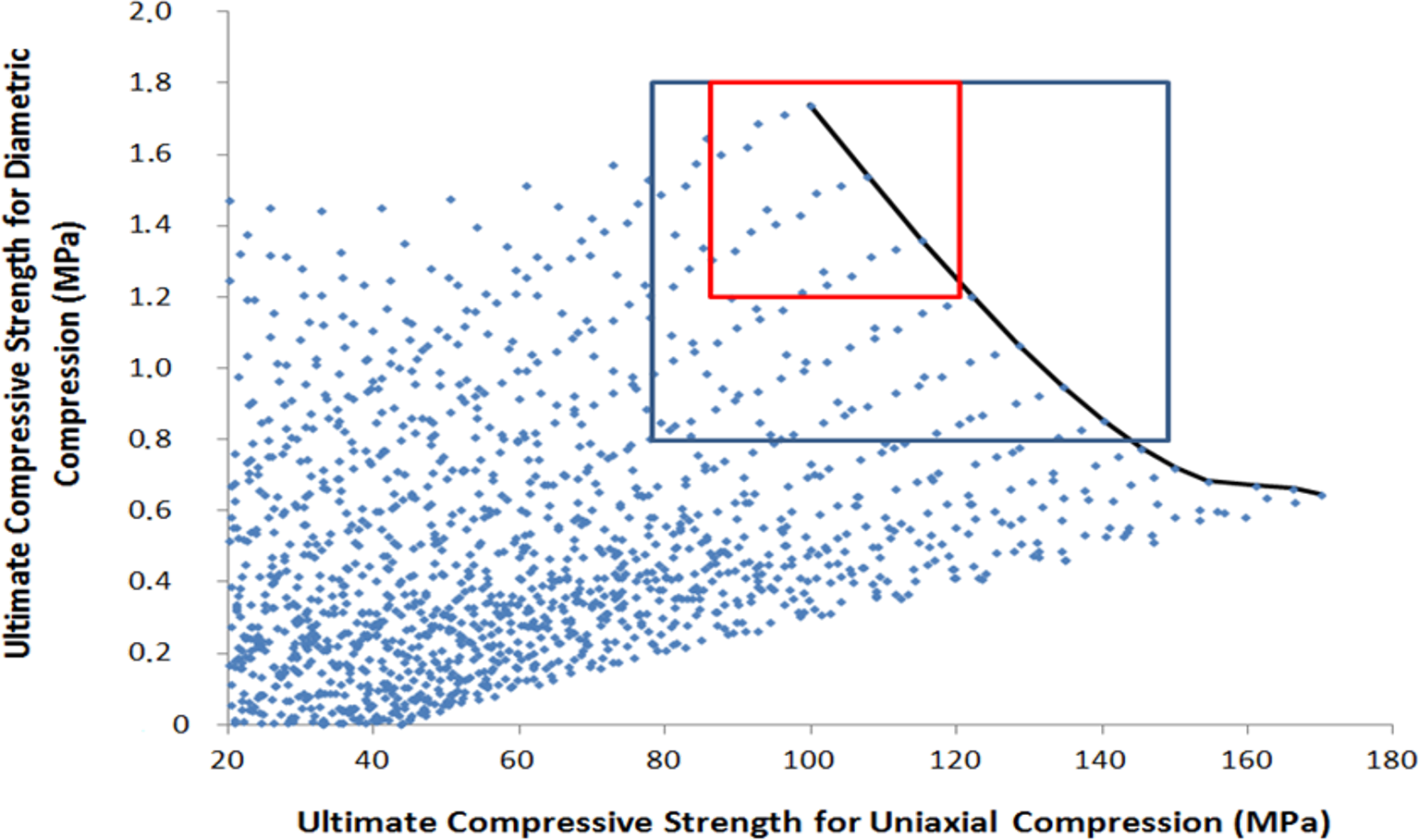
Operating points in a *Y* (MPa) vs *Z* (MPa) diagram. *Y*(*Z*) is the ultimate compressive strength for diametric (uniaxial) compression. The black curve is called the Pareto curve. The red square is the selection of the best pellets; *Y* ≥ 90 MPa and *Z* ≥ 1.2 MPa. The blue square represents a larger selection of the optimum pellets; *Y* ≥ 80 MPa and *Z* ≥ 0.8 MPa.

The parameter analyses performed in previous sections (uniaxial and diametric compression tests) have shown the optimal pelletization conditions to have the best mechanical properties with respect to compression tests. With these two equations, the optimal dimensions and conditions can be found in order to form pellets with the highest mechanical properties (in terms of uniaxial and diametric compression tests). Figure 8 shows all the operating points calculated by Equation 1 and Equation 2 in a diametric ultimate compression strength vs uniaxial ultimate compression strength diagram.

The optimal operating points (Pareto curve) are depicted by the black curve. This curve is the result of the best couples (*Y*,*Z*) [34]. These theoretical results are interesting but exclude an interesting part of the operating points from a spatial application point of view. Moreover, a part of the curve leads to low mechanical properties in terms of diametric compression tests. To have a better selection of pellets, two different scenarios are studied. The first one (red square) is the selection of the best pellets; *Y* ≥ 90 MPa and *Z* ≥ 1.2 MPa. A second selection (blue square) represents a larger selection of the optimum pellets; *Y* ≥ 80 MPa and *Z* ≥ 0.8 MPa. These two scenarios ensure high mechanical strength pellets with a wide range of pellet sizes at the same time.

These results have independently shown that the best mechanical performance is reached for high applied pressures and thin pellets. On the contrary, the pellet diameter improves uniaxial mechanical properties but weakens the pellet during diametric compression tests. To estimate the optimal range of the pelletization conditions, Equation 1 and Equation 2 are simultaneously used to calculate parameters relative to the selection of operating points.

**Figure 9:**
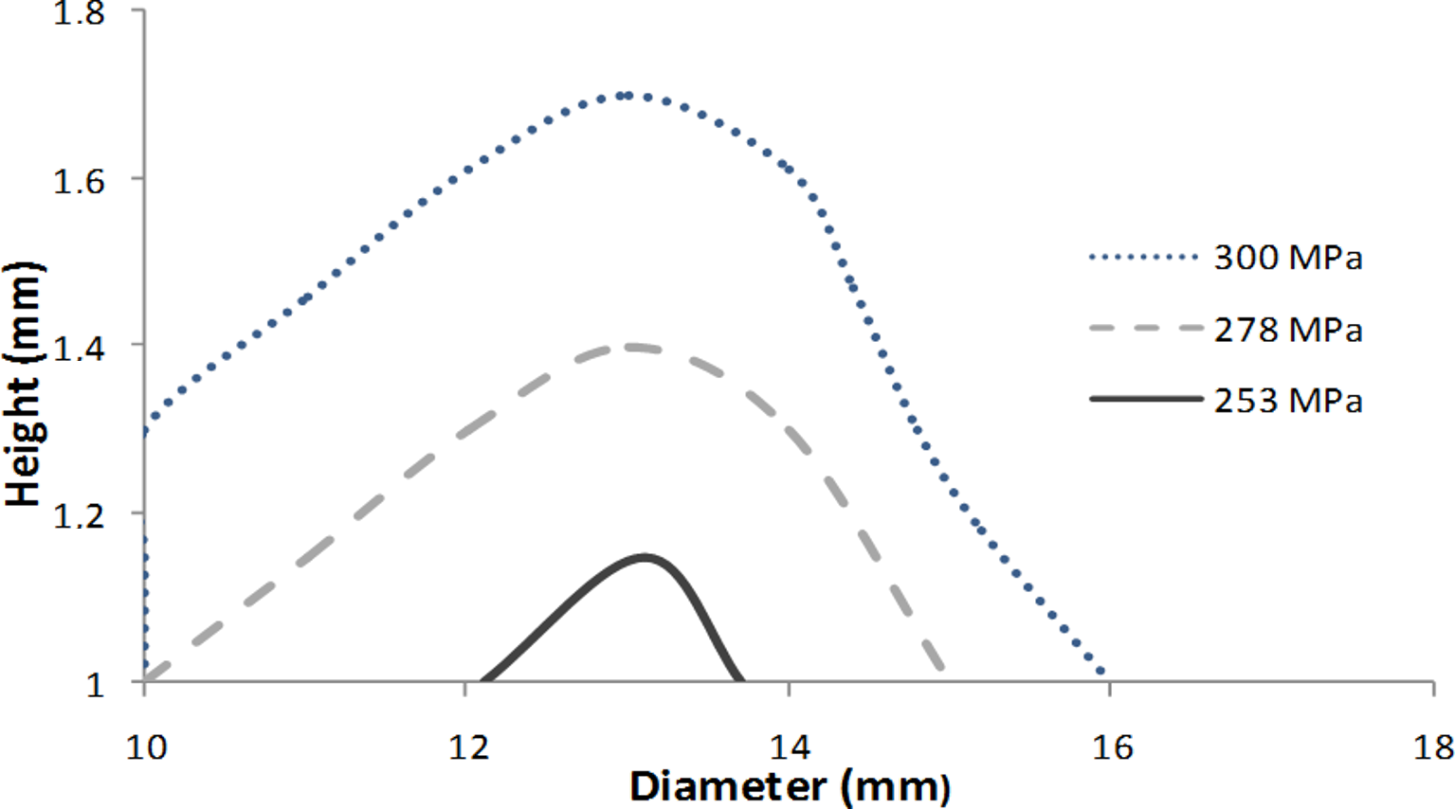
Pellet size and applied pressure for the best mechanical properties; scenario 1 (red square in Figure 8) is the selection of the best pellets with *Y* ≥ 90 MPa and *Z* ≥ 1.2 MPa.

Figure 9 shows the operating parameters (diameter, height and applied pressure) corresponding to the best operating point (red square).

**Figure 10:**
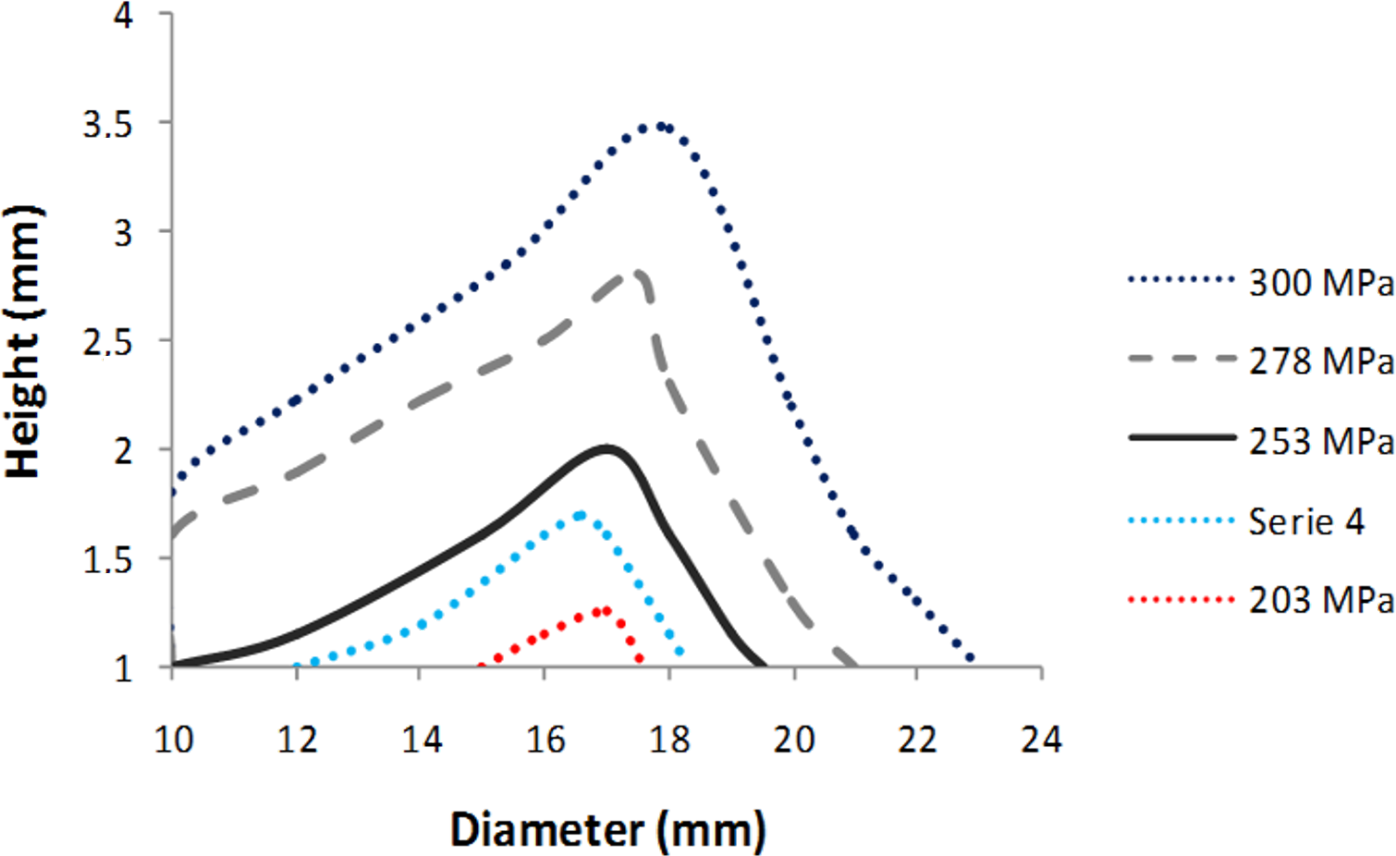
Pellet size and applied pressure for the best mechanical properties; scenario 2 (blue square in Figure 8) is the selection of the best pellets with *Y* ≥ 80 MPa and *Z* ≥ 0.8 MPa.

The operating optimums are obtained for high applied pressure, thin pellets (less than 1.7 mm) with a diameter less than 16 mm. The largest selection of operating conditions is obtained for pellet diameters between 12 and 14 mm. As previously observed, the most dense and compact pellets have the best mechanical properties. Figure 10 shows the operating parameters (diameter, height and applied pressure) corresponding to the best operating points of scenario 2 (blue square).

Many options of pellet sizes (height and diameter) are possible. The operating optimums are obtained for high applied pressure, thin pellets (up to 3.5 mm) and in a wide range of diameters (from 10 to 23 mm).

## Conclusion

Two empirical models were developed allowing the estimation of the mechanical properties of all zeolite pellets made with 5 wt % binder (methylcellulose or sodium metasilicate). Two mechanical tests were carried out: uniaxial and diametric compressions. The optimum pellet dimensions are a diameter in the range of 10–23 mm, a height of 1–3.5 mm and an applied pressure higher than 200 MPa. Experimental values may of course be out these defined domains but the presented model will not predict the mechanical properties of them.

These pellets can be used in molecular decontamination applications, especially in the aerospace field where the dimensions are very important. Indeed, the available space inside a satellite is limited, and the dimensions of any adsorbent bodies have to be controlled to avoid increased costs of the satellite. This methodology allows the various dimensions of pellets while respecting technical constraints and at the same time ensuring good mechanical properties. The adsorption properties of the zeolite pellets were also preserved [8].
